# A Fortified Method to Screen and Detect Left Ventricular Hypertrophy in Asymptomatic Hypertensive Adults: A Korean Retrospective, Cross-Sectional Study

**DOI:** 10.1155/2018/6072740

**Published:** 2018-11-25

**Authors:** Hyo Eun Park, Sung-Bin Chon, Sang Hoon Na, Heesun Lee, Su-Yeon Choi

**Affiliations:** ^1^Department of Internal Medicine, Seoul National University Hospital, Seoul, Republic of Korea; ^2^Department of Internal Medicine, Healthcare System Gangnam Center, Seoul National University Hospital, Seoul, Republic of Korea; ^3^Department of Emergency Medicine, CHA Bundang Medical Center, CHA University, Gyeonggi-Do, Republic of Korea; ^4^Department of Emergency Medicine, Seoul National University Hospital, Seoul, Republic of Korea

## Abstract

**Purpose:**

Left ventricular (LV) mass is determined by the wall thickness and diameter. LV hypertrophy (LVH), the increase in LV mass, is usually screened with electrocardiography but is often insensitive. We tried to fortify the rule to detect LVH using cardiothoracic ratio (CTR) in chest X-ray and well-known risk factors besides electrocardiography.

**Materials and Methods:**

This retrospective cross-sectional study included asymptomatic hypertensive individuals aged ≥40 y who underwent voluntary checkups including echocardiography. Independent variables to explain LVH (LV mass index>115 g/m^2^ for men and >95 g/m^2^ for women calculated on echocardiography) were chosen among Sokolow-Lyon voltage amplitude (SLVA), CTR and cardiovascular risk factors by multiple logistic regression analysis. The diagnostic rule to detect LVH was made by summing up the rounded-off odds ratio of each independent variable and was validated using bootstrapping method.

**Results:**

Among the 789 cases enrolled (202 females (25.6%), mean age 59.6±8.8 y), 168 (21.3%) had LVH. The diagnostic rule summed female, age≥65 y, BMI≥25 kg/m^2^, SLVA≥35 mm, and CTR≥0.50 (scoring 1 per each). Its c-statistics was 0.700 (95% CI: 0.653, 0.747), significantly higher (*p*<0.001) than that of SLVA≥35 mm, 0.522 (95% CI: 0.472, 0.572). The sensitivity and specificity of the model were 61.9% and 72.1% for score≥2 and 30.4% and 92.9% for score≥3. The SLVA≥35 mm criteria showed sensitivity of 12.5% and specificity of 91.9%.

**Conclusions:**

The rule to sum up the number of the risk factors of female, age≥65 y, BMI≥25 kg/m^2^, SLVA≥35 mm, and CTR≥0.50 may be a better diagnostic tool for screening LVH, than the electrocardiography-only criteria, at the score≥2.

## 1. Introduction

Left ventricular hypertrophy (LVH), an increase of left ventricular (LV) mass, is common in hypertensive patients and increases the risk of sudden cardiac death, cerebrovascular events, heart failure, death following myocardial infarction, and arrhythmias [1–7]. The regression of LV mass index is associated with lower incidence of cardiovascular events and improved cardiac function [8–12], and thus finding subjects at risk before clinical symptom appears is important in terms of disease prevention.

Transthoracic echocardiography is the current “gold standard” to accurately measure LV mass and confirm LVH [13–15]. Despite the advantages of echocardiography as a noninvasive imaging modality which can be performed at bedside and without radiation exposure, echocardiography is not an appropriate method for public screening tool. It is expensive, time-consuming, and expert-dependent to be used as a screening method. Instead, electrocardiography (ECG) criteria have been used as screening tools to detect LVH in asymptomatic subjects.

Enlarged cardiothoracic ratio (CTR), defined as >0.50, is another parameter to determine cardiac enlargement, which can be easily measured from chest X-ray. It is the most widely known chest radiograph index of cardiac function. Enlarged CTR, defined as >0.50, has been evaluated in patients with chronic kidney disease under hemodialysis and has shown prognostic significance [16, 17]. Both CTR and ECG can be easily obtained quickly and without use of contrast agent and potentially can be used as initial screening methods for large number of subjects [18, 19].

In current study, we evaluated diagnostic value of CTR, ECG criteria, and the well-known risk factors of LVH and tried to develop a fortified rule to screen LVH combining them, to be used in primary clinics and in real-world public population.

## 2. Materials and Methods

### 2.1. Study Subjects

The cross-sectional study was conducted retrospectively. Random samples of subjects were taken from the subjects who had healthcare check-up at Healthcare System Gangnam Center, Seoul National University Hospital. All included subjects were hypertensive patients under management or newly detected hypertensive subjects of age≥40 years, who had chest X-ray, ECG, and echocardiography within one month of the medical check-up.

Exclusion criteria were as follows: (1) missing data among any one of following: chest X-ray, ECG, or echocardiography; (2) indeterminate cardiac diameter (CD) on chest X-ray due to various reasons [20]; (3) bundle branch blocks with inappropriate S or R waves to calculate ECG-based LVH criteria [18, 19, 21]; (4) inability to calculate LV mass from echocardiography due to poor imaging window; and (5) any known significant ischemic or valvular heart disease, any type of cardiomyopathy or infiltrative disorders. From 836 subjects initially screened, 47 subjects were excluded and in final study analysis 789 hypertensive patients were included.

The study protocol was approved by the Institutional Review Board of Seoul National University Hospital and followed the ethical guidelines of the Declaration of Helsinki as revised in 2013 (IRB No. H-1405-001-573). Due to the retrospective design using a database and medical records, informed consent was waived by the board.

### 2.2. Methods of Measurement, Data Collection, and Processing

Basic demographic characteristics included age, gender, height, weight, body mass index (BMI), systolic blood pressure (SBP), and diastolic blood pressure (DBP). Height and body weight were measured using a digital scale. BMI was calculated using height and weight according to the formula: BMI=weight (kg)/height (m)^2^. Based on the subject-recorded questionnaires and medications, presence of comorbid conditions such as diabetes mellitus and hyperlipidemia was screened [22].

The laboratory tests were taken after fasting for at least 12 hours. Blood tests included total cholesterol, triglyceride, high-density lipoprotein (HDL) cholesterol, low-density lipoprotein (LDL) cholesterol, fasting blood sugar, glycated hemoglobin, blood urea nitrogen, and serum creatinine level.

**Figure 1:**
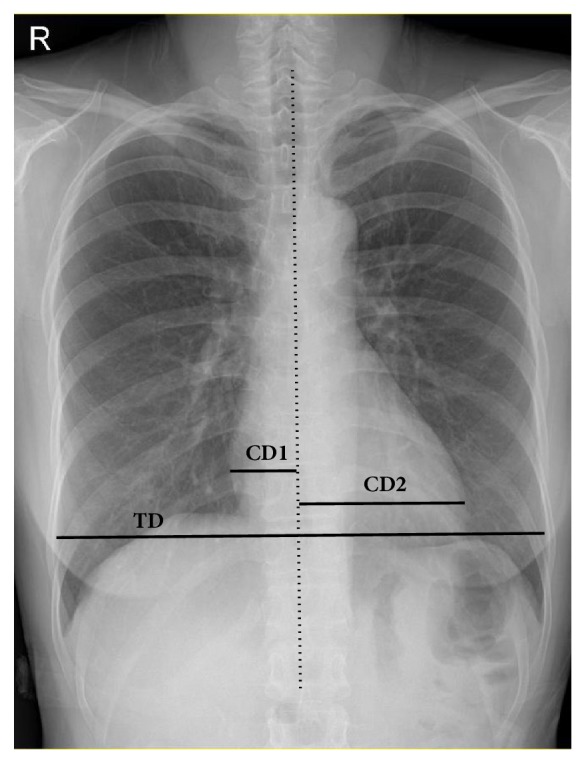
**Measurement of cardiac diameter (CD) and thoracic diameter (TD)**. On chest PA, a vertical line (dotted line) was traced parallel to the vertebral column. The greatest distances from this line to each cardiac border (CD 1 and CD 2) were summed up to get CD. TD was defined as the greatest width (TD) between the inner surfaces of ribs. CD, cardiac diameter; TD, thoracic diameter; chest PA, posteroanterior chest X-ray.

To measure CD and CTR on chest X-ray, a vertical line was traced parallel to the vertebral column and the greatest distances from the vertical line to each cardiac border were summed. Thoracic diameter (TD) was defined as the greatest width between the inner surfaces of ribs (Figure 1). CTR was calculated by CD/TD [20].

To evaluate LVH from ECG, two different criteria were used. The tallest heights of S wave in V1 and R wave in V5 or 6 were summed to render Sokolow-Lyon voltage amplitude (SLVA) [18, 19], and SLVA≥35 mm was used to define LVH [23]. With the sum of R wave in aVL and S wave in V3 set as Cornell voltage amplitude, CVA≥20 mm for women and 28mm for men were applied to define LVH by Cornell voltage criteria [24].

Echocardiographic measurement was used to calculate LV mass. LVH was defined when LV mass indexed by body surface area (BSA) was ≥115 g/m^2^ for male and ≥95 g/m^2^ for female subjects, respectively [13, 25]. LV mass was calculated with the linear method using echocardiography performed by experienced cardiologists:

(1) LV  mass  g=0.8×1.04×LVID+LVPWT+IVST3-LVID3+0.6

where LVID indicates LV internal diameter, LVPWT the LV posterior wall thickness, and IVST the interventricular septal thickness [13, 14]. LV dimensions and wall thickness were measured using M-mode. BSA (m^2^) was calculated as ‘√height (cm)x√weight (kg)/60 [26].

To minimize interrater variability, one investigator (PHE) abstracted all data of SVLA and CTR and another (NSH) verified interrater reliability by reviewing 3% of them chosen randomly.

### 2.3. Data Analysis

To show demographic characteristics and comorbidity, the mean and standard deviation (SD) of continuous variables and proportions of categorical values were reported. Using the t-test and chi-square test, the candidate variables to show differences between those with and without LVH were identified. The cut-off point of the* P* value was <0.20, here. Among these, continuous variables were converted into categorical ones according to spline analysis. When appropriate, well-known cut-off values were preferred.

Incorporating the chosen variables, we performed multiple logistic regression analysis by conditional forward selection to identify independent risk factors of LVH. To build an easy-to-use diagnostic rule, the authors multiplied the odds ratio (OR) of each risk factor by an arbitrary number and rounded the results to the nearest integers. The diagnostic index was defined as the sum of the corresponding simplified OR's. The discrimination accuracy to detect LVH was evaluated by calculating the area under the ROC curve and compared with that of the traditional Sokolow-Lyon criteria of LVH by the method suggested by DeLong et al. [27]. All the assumptions required for logistic regression analysis were verified.

Afterwards, this diagnostic rule was validated internally by bootstrapping method with 1,000 repetitions to show the corrected area under the curve (AUC) [28].

IBM SPSS Statistics (SPSS Inc., Chicago, IL, USA) Version 24 and R (R Foundation for Statistical Computing, Vienna, Austria [http://www.R-project.org]) with the POCR, pROC, and verification packages (http://cran.r-project.org) were used in the analyses. A two-sided* p*<0.05 was used to determine statistical significance unless described otherwise.

## 3. Results

The mean age of the 789 subjects was 56.9 years (SD, 8.8 years) and 202 subjects were females (25.6%). Diabetes mellitus was present in 136 subjects (17.2%) and dyslipidemia was present in 278 subjects (35.2%). LVH was detected in 168 subjects (21.3%) by echocardiography, which was more prevalent in female gender (15.0% versus (vs.) 39.6% in male vs. females, p<0.001) [29, 30]. Electrocardiographically diagnosed LVH by SLVA ≥35 mm was present in 71 subjects (9.0%), of whom 21 subjects had LVH with echocardiography diagnosis. By Cornell voltage criteria, LVH was present in 40 subjects of whom 13 subjects had LVH by echocardiography. CTR ≥0.50 was present in 157 subjects (19.9%), of whom 58 subjects had echocardiography finding of LVH. The intraclass correlation coefficients in the measurement of CD, TD, CTR, and SLVA were 0.962, 0.993, 0.960, and 0.983, respectively (n=27, all* p*<0.001).

The study subjects were grouped into LVH group and control group according to the presence of LVH diagnosed by echocardiography. Compared to control group, LVH group had significantly greater number of female subjects, older age, higher SBP and DBP, and greater SLVA in electrocardiogram (Table 1). Among the candidate variables which showed differences (*p*>0.20 at this stage) between LVH and control groups, SBP and CTR were chosen rather than DBP and CD, respectively, considering collinearity and clinical importance. Before the logistic regression analysis, the continuous variables were categorized as age≥65 vs. <65 y, height≤1.65 m vs. >1.65 m, weight≤67 vs. >67 kg, BMI≥25 vs. <25 kg/m^2^, SBP≥140 vs. <140mmHg, HDL cholesterol≥1.55 vs. <1.55mmol/L, LDL cholesterol≤2.59 vs. >2.59 mmol/L, BUN≥7.14 vs. <7.14 mmol/L, CTR≥0.5 vs. <0.5, and SLVA≥35 vs. <35mm. Since the study aim was focused on public screening, the echocardiographic variables were not taken into consideration.

After univariate logistic regression analysis, step-wise multiple logistic regression analysis was performed with these candidate variables to detect LVH. Female gender, age≥65 years, BMI≥25 kg/m^2^, CTR≥0.50, and SLVA≥35 mm were chosen as the independent predictors of LVH (Table 2). Hosmer-Lemeshow goodness-of-fit was satisfied (*p*=0.681). When arbitrary number of 0.39 was multiplied to the odds ratio of each predictor and rounded up, the simplified score was 1 for each (Table 2).

The five variables described in Table 2 were used to model a new score system to detect LVH. Each variable was given one point, and higher score showed greater association with presence of LVH. The OR for LVH was 2.755 (95% CI: 1.614-4.701) for score ≥1, 4.208 (95% CI: 2.944-6.016) for score≥2, 5.716 (95% CI: 3.646-8.961) for score≥3, and 6.432 (95% CI: 2.862-14.455) for score≥4 (all* p*<0.001). No case scored 5.

**Figure 2:**
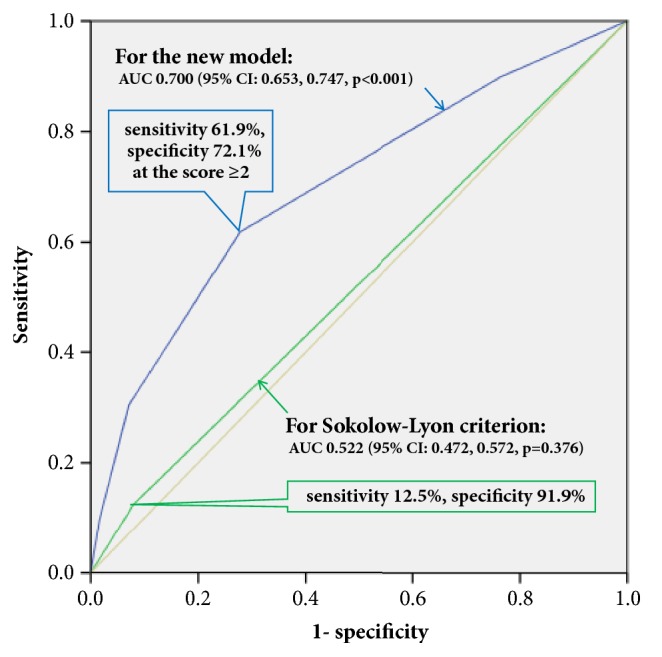
**The receiver operating characteristic curves of the new model and the traditional Sokolow-Lyon criterion to detect left ventricular hypertrophy (LVH)**. The new model is the sum of the number of following risk factors: age≥65 y, female, BMI≥25 kg/m^2^, SLVA≥35 mm, and CTR≥0.50. The Sokolow-Lyon criterion is positive when the Sokolow-Lyon voltage amplitude is ≥35 mm. AUC, area under curve; CI, confidence interval.

The area under the ROC curve of this model was 0.700 and was internally validated by bootstrapping (95% CI: 0.655, 0.745,* p*<0.001), while that for the traditional criterion of SLVA≥35 mm was 0.522 (95% CI: 0.472, 0.572,* p*=0.376) (Figure 2). Meanwhile, those for female gender, age≥65 years, BMI≥25 kg/m^2^ and CTR≥0.50 were 0.640 (95% CI: 0.590, 0.690,* p*<0.001), 0.613 (95% CI: 0.563, 0.663,* p*<0.001), 0.525 (95% CI: 0.476, 0.575,* p*=0.311), and 0.593 (95% CI: 0.542, 0.644, p*<*0.001), respectively. Thus, the areas differed significantly between the new model and the rule of SLVA≥35 mm (0.178, 95% CI: 0.130, 0.226,* p*<0.001). The sensitivity and specificity of this new model were 61.9% and 72.1% for score≥2 and 30.4% and 92.9% for score≥3. Meanwhile, the traditional SLVA≥35 mm criteria showed sensitivity of 12.5% and specificity of 91.9% (Table 3).

## 4. Discussion

Our study suggested a new scoring system to determine LVH more accurately, which includes clinical, radiologic, and electrical information. According to our knowledge, this is the first study to build a scoring system by combining various clinical risk factors of LVH, CTR in chest X-ray, and SLVA in ECG. Although there have been studies to modify diagnostic accuracy of ECG, none had presented a scoring system including clinically significant parameters [16, 17]. With utilization of the well-known clinical risk factors, traditional ECG diagnostic criteria of LVH and CTR≥0.50, we could build a relatively clear-cut and easy-to-use model to screen LVH far better than the traditional ECG criteria [31]. Considering the sensitivity and specificity, score≥2 could be used for screening cut-off value of LVH. Meanwhile, at the score≥3, LVH could be specifically suggested (Table 3).

### 4.1. Screening LVH by ECG: Advantages and Limitations to Overcome

As ECG is not costly and simple to perform, it is widely used to determine LVH in clinical practice and still remains to be the most commonly used screening tool [32, 33]. Despite the advantages, poor diagnostic accuracy and low sensitivity of ECG criteria limit its use in detecting LVH [34, 35], and there have been many studies to “adjust” ECG criteria to improve diagnostic accuracy for detection of LVH [36]. For example, Rider et al. reported obesity results in decrease of voltage amplitude and leftward shift in anatomical axis, thereby causing even poorer sensitivity and diagnostic accuracy of ECG [36].

### 4.2. Screening LVH by Chest X-Ray: Use of CTR

Chest X-ray is another commonly used diagnostic tool. The cardiac silhouette is often evaluated to determine whether there is chamber enlargement, and CTR of 50% from chest X-ray has been considered as a cut-off value reflecting LV enlargement [37]. From a pooled analysis including 466 patients, CTR alone had 83.3% sensitivity, 45.4% specificity, 43.5% positive predictive value, and 82.7% negative predictive value [17], making CTR neither valuable as a screening nor a confirmatory test. There also has been a study showing increase of CTR by 2.0% over 9 years of follow-up, although clinical significance had been questioned [38]. Increase of CTR in elderly is due to not only increase in cardiac size but also decrease in thoracic diameter, which is associated with aging. Moreover, CTR has also failed to show strong predictive value or correlation with LV dysfunction [39, 40].

### 4.3. Screening LVH by Echocardiography and Cardiovascular Magnetic Resonance (CMR): Advantages and Limitations to Overcome

Imaging modalities such as transthoracic echocardiography [41] and CMR are accurate determinants of LVH, and their accuracy exceeds that of ECG. However, such imaging modalities are not always available, are difficult to operate, and are also expensive, which limit the wide use as public screening tools. Rather, these imaging modalities can give definite diagnosis and quantitative measurements.

### 4.4. Study Limitation

Since our study subjects include a narrow spectrum of asymptomatic hypertensive patients, it should not be extrapolated to general population before further evaluation. The new scoring system we proposed here needs further validation in mass population, and the diagnostic performance should be compared to that of classical modalities. The new scoring system as a prognosticator should also be evaluated, since this study did not evaluate prognosis and outcome. Nevertheless, given poor diagnostic value of ECG as a single parameter to determine LVH and poor accessibility to imaging modality for public screening method, our new scoring system allows simple and readily available assessment to determine LVH.

To conclude, the new scoring system from our study allows simple and readily available assessment to determine LVH. This simple scoring system significantly improved the power of ECG or CTR to detect LVH. Improving diagnostic accuracy allows early detection of LVH, which eventually will help reducing end-organ damage and other complications related to LVH, especially in the perspective of primary care and public health. Although not studied yet, it may eventually help reduce adverse events associated with LVH.

## Figures and Tables

**Table 1:** Baseline characteristics of study subjects according to the presence or absence of echocardiographic left ventricular hypertrophy by LV mass.

	LVH (n=168)	No LVH (n=621)	*p* value
Demographic information			
Female, n (%)	80 (47.6%)	122 (19.6%)	<0.001^*∗∗*^
Age, years	64 ± 10	59 ± 8	<0.001^*∗∗*^
Diabetes mellitus, n (%)	32 (19.0%)	104 (16.7%)	0.558
Hyperlipidemia, n (%)	61 (36.3%)	217 (34.9%)	0.812
Height, m	1.63 ± 0.09	1.67 ± 0.07	<0.001^*∗∗*^
Weight, kg	67.5 ± 11.9	70.1 ± 11.2	0.007^*∗∗*^
BMI, kg/m^2^	25.3 ± 3.1	24.9 ± 2.8	0.058
SBP, mmHg	126.4 ± 13.2	122.5 ± 13.5	0.001^*∗∗*^
DBP, mmHg	79.7 ± 9.5	81.4 ± 9.8	0.045^*∗*^
Laboratory results			
Fasting glucose, mmol/L	5.83 ± 1.00	5.88 ± 1.11	0.422
HbA1c, %	5.9 ± 0.5	5.9 ± 0.7	0.342
Total cholesterol, mmol/L	4.66 ± 0.91	4.74 ± 0.88	0.335
Triglyceride, mmol/L	1.39 ± 0.75	1.47 ± 0.87	0.295
HDL cholesterol, mmol/L	1.40 ± 0.34	1.32 ± 0.28	0.012^*∗*^
LDL cholesterol, mmol/L	2.75 ± 0.75	2.90 ± 0.75	0.009^*∗∗*^
BUN, mmol/L	6.07 ± 2.14	5.71 ± 1.78	0.063
Cr, *μ*mol/L	79.56 ± 17.68	79.56 ± 35.36	0.359
Echocardiography measurement			
LVIDd, mm	52 ± 4	48 ± 4	<0.001^*∗∗*^
LVIDs, mm	30 ± 4	28 ± 3	<0.001^*∗∗*^
LVEF, %	67 ± 6	67 ± 5	0.943
IVSd, mm	11 ± 1	9 ± 1	<0.001^*∗∗*^
LVPWd, mm	11 ± 1	9 ± 1	<0.001^*∗∗*^
LV mass, g	213 ± 43	159 ± 33	<0.001^*∗∗*^
LV mass/BSA, g/m^2^	122 ± 18	88 ± 14	<0.001^*∗∗*^
Radiology measurement			
CD, mm	139.4 ± 14.3	137.1 ± 14.2	0.067
CTR	0.48 ± 0.05	0.46 ± 0.04	<0.001^*∗∗*^
Electrocardiography measurement			
SLVA, mm	25.7 ± 8.9	24.1 ± 7.2	0.042^*∗*^
CVA, mm	15.5 ± 5.8	15.0 ± 5.8	0.313

BMI, body mass index; BSA, body surface area; BUN, blood urea nitrogen; CD, cardiac diameter; Cr, serum creatinine; CTR, cardiothoracic ratio; CVA, Cornell voltage amplitude; DBP, diastolic blood pressure; HbA1c, glycated hemoglobin; HDL, high-density lipoprotein; IVSd, interventricular septum end-diastolic thickness; LV, left ventricle; LVEF, left ventricular ejection fraction; LVH, left ventricular hypertrophy; LVIDd, left ventricular end-diastolic diameter; LVIDs, left ventricular end-systolic diameter; LVPWd, left ventricular end-diastolic posterior wall thickness; LDL, low-density lipoprotein; SBP, systolic blood pressure; SLVA, Sokolow-Lyon voltage amplitude

^*∗*^ *p* <0.05

^*∗∗*^ *p* <0.01.

**Table 2:** Logistic regression analyses to reveal the predictors of LVH by LV mass using echocardiography and the relevant simplified scores.

	OR	95% CI	*p*	Score^*∗*^ (for multivariate analysis only)
Univariate logistic regression analysis				
Female	3.718	2.590-5.339	<0.001	N/A
Age≥65 years	2.838	1.985-4.057	<0.001	N/A
Height≤1.65 m	2.807	1.980-3.978	<0.001	N/A
Weight≤67 kg	1.838	1.303-2.591	0.001	N/A
BMI≥25 kg/m^2^	1.226	0.872-1.724	0.242	N/A
SBP≥140 mmHg	1.996	1.241-3.209	0.004	N/A
HDL cholesterol≥1.55 mmol/L	1.541	1.059-2.242	0.024	N/A
LDL cholesterol≤2.59 mmol/L	1.449	1.026-2.048	0.035	N/A
BUN≥7.14 mmol/L	1.618	1.061-2.469	0.025	N/A
CTR≥0.50	2.780	1.894-4.081	<0.001	N/A
SLVA≥35 mm	1.631	0.950-2.802	0.076	N/A
Multivariate logistic regression analysis to detect LVH				
Female	3.544	2.370-5.299	<0.001	1
Age≥65 years	2.205	1.500-3.241	<0.001	1
BMI≥25 kg/m^2^	1.591	1.084-2.337	0.018	1
CTR≥0.50	1.774	1.163-2.707	0.008	1
SLVA≥35 mm	2.205	1.231-3.950	0.008	1

BMI, body mass index; BUN, blood urea nitrogen; CI, confidence interval; CTR, cardiothoracic ratio; HDL, high-density lipoprotein; LDL, low-density lipoprotein; LVH, left ventricular hypertrophy; N/A, not applicable; OR, odds ratio; SBP, systolic blood pressure; SLVA, Sokolow-Lyon voltage amplitude.

^*∗*^To build an easy-to-use screening rule to detect LVH, the score was rendered by multiplying the OR by an arbitrary number of 0.39 and rounding it up.

**Table 3:** Diagnostic accuracy of the new model to detect left ventricular hypertrophy by echocardiography according to the scores of the new system.

Score^*∗*^	Sensitivity	Specificity	LR (+)	LR (-)
≥1	89.9%	23.7%	1.18	0.43
≥2	61.9%	72.1%	2.22	0.53
≥3	30.4%	92.9%	4.28	0.75
≥4	9.5%	98.4%	5.94	0.92

BMI, body mass index; CTR, cardiothoracic ratio; LR (+), positive likelihood ratio; LR (-), negative likelihood ratio; SLVA, Sokolow-Lyon voltage amplitude.

^*∗*^Age≥65 y, female, BMI≥25 kg/m^2^, SLVA≥35 mm, and CTR≥0.50 were scored 1 for each.

## Data Availability

The original raw data used to support the findings of this study are restricted by the Institutional Review Board of Seoul National University Hospital and Healthcare Research Institute, Gangnam Center, in order to protect patient privacy. Data are available from the corresponding author (Sang-Hoon Na, nasanghoon@gmail.com) for researchers who meet the criteria for access to confidential data.
